# Fitting Splines to Axonal Arbors Quantifies Relationship Between Branch Order and Geometry

**DOI:** 10.3389/fninf.2021.704627

**Published:** 2021-08-11

**Authors:** Thomas L. Athey, Jacopo Teneggi, Joshua T. Vogelstein, Daniel J. Tward, Ulrich Mueller, Michael I. Miller

**Affiliations:** ^1^Institute for Computational Medicine, Johns Hopkins University, Baltimore, MD, United States; ^2^Department of Biomedical Engineering, Johns Hopkins University, Baltimore, MD, United States; ^3^Center for Imaging Science, Johns Hopkins University, Baltimore, MD, United States; ^4^Kavli Neuroscience Discovery Institute, Johns Hopkins University, Baltimore, MD, United States; ^5^Department of Computational Medicine, University of California, Los Angeles, Los Angeles, CA, United States; ^6^Department of Neurology, University of California, Los Angeles, Los Angeles, CA, United States; ^7^Department of Neuroscience, Johns Hopkins University, Baltimore, MD, United States

**Keywords:** neuron, morphology, axon, curvature, projection, mouse, spline, python

## Abstract

Neuromorphology is crucial to identifying neuronal subtypes and understanding learning. It is also implicated in neurological disease. However, standard morphological analysis focuses on macroscopic features such as branching frequency and connectivity between regions, and often neglects the internal geometry of neurons. In this work, we treat neuron trace points as a sampling of differentiable curves and fit them with a set of branching B-splines. We designed our representation with the Frenet-Serret formulas from differential geometry in mind. The Frenet-Serret formulas completely characterize smooth curves, and involve two parameters, curvature and torsion. Our representation makes it possible to compute these parameters from neuron traces in closed form. These parameters are defined continuously along the curve, in contrast to other parameters like tortuosity which depend on start and end points. We applied our method to a dataset of cortical projection neurons traced in two mouse brains, and found that the parameters are distributed differently between primary, collateral, and terminal axon branches, thus quantifying geometric differences between different components of an axonal arbor. The results agreed in both brains, further validating our representation. The code used in this work can be readily applied to neuron traces in SWC format and is available in our open-source Python package brainlit: http://brainlit.neurodata.io/.

## 1. Introduction

Not long after scientists like Ramon y Cajal started studying the nervous system with staining and microscopy, neuron morphology became a central topic in neuroscience (Parekh and Ascoli, 2013). Morphology became the obvious way to organize neurons into categories such as pyramidal cells, Purkinje cells, and stellate cells. However, morphology is important not only for neuron subtyping, but in understanding learning and disease. For example, a now classic neuroscience experiment found altered morphology in geniculocortical axonal arbors in kittens whose eyes had been stitched shut upon birth (Antonini and Stryker, 1993). Also, morphological changes have been associated with the gene underlying an inherited form of Parkinson's disease (MacLeod et al., 2006). Neuron morphology has been an important part of neuroscience for over a century, and remains so – one of the BRAIN Initiative Cell Census Network's primary goals is to systematically characterize neuron morphology in the mammalian brain.

Currently, studying neuron morphology typically involves imaging one or more neurons, then tracing the cells and storing the traces in a digital format. Several recent initiatives have accumulated large datasets of neuron traces to facilitate morphology research. NeuroMorpho.Org, for example, hosts a total of over 140,000 neuron traces from a variety of animal species (Ascoli et al., 2007). These traces are typically stored as a list of vertices, each with some associated attributes including connections to other vertices.

Many scientists analyze neuron morphology by computing various summary features such as number of branch points, total length, and total encompassed volume. Neurolucida, a popular neuromorphology software, employs this technique. Another approach focuses on neuron topology, and uses metrics such as tree edit distance (Heumann and Wittum, 2009). However, both of these approaches neglect *kinematic* geometry, or how the neuron travels through space. Tortuosity index is a summary feature that captures internal axon geometry, but this feature depends on the definition of start and end points, and cannot capture an axon's curvature at a single point.

In this work, we look at neuron traces through the lens of differential geometry. In particular, we establish a system of fitting interpolating splines to the neuron traces, and computing their curvature and torsion properties. To our knowledge, curvature and torsion have never been measured in neuron traces. We applied this method to cortical projection neuron traces from two mouse brains in the MouseLight dataset from HHMI Janelia (Winnubst et al., 2019). In both brains, we found different distributions of these properties between primary, collateral, and terminal axon segments. The code used in this work is available in our open-source Python package brainlit: http://brainlit.neurodata.io/.

## 2. Methods

### 2.1. Spline Fitting

**Figure 1:**
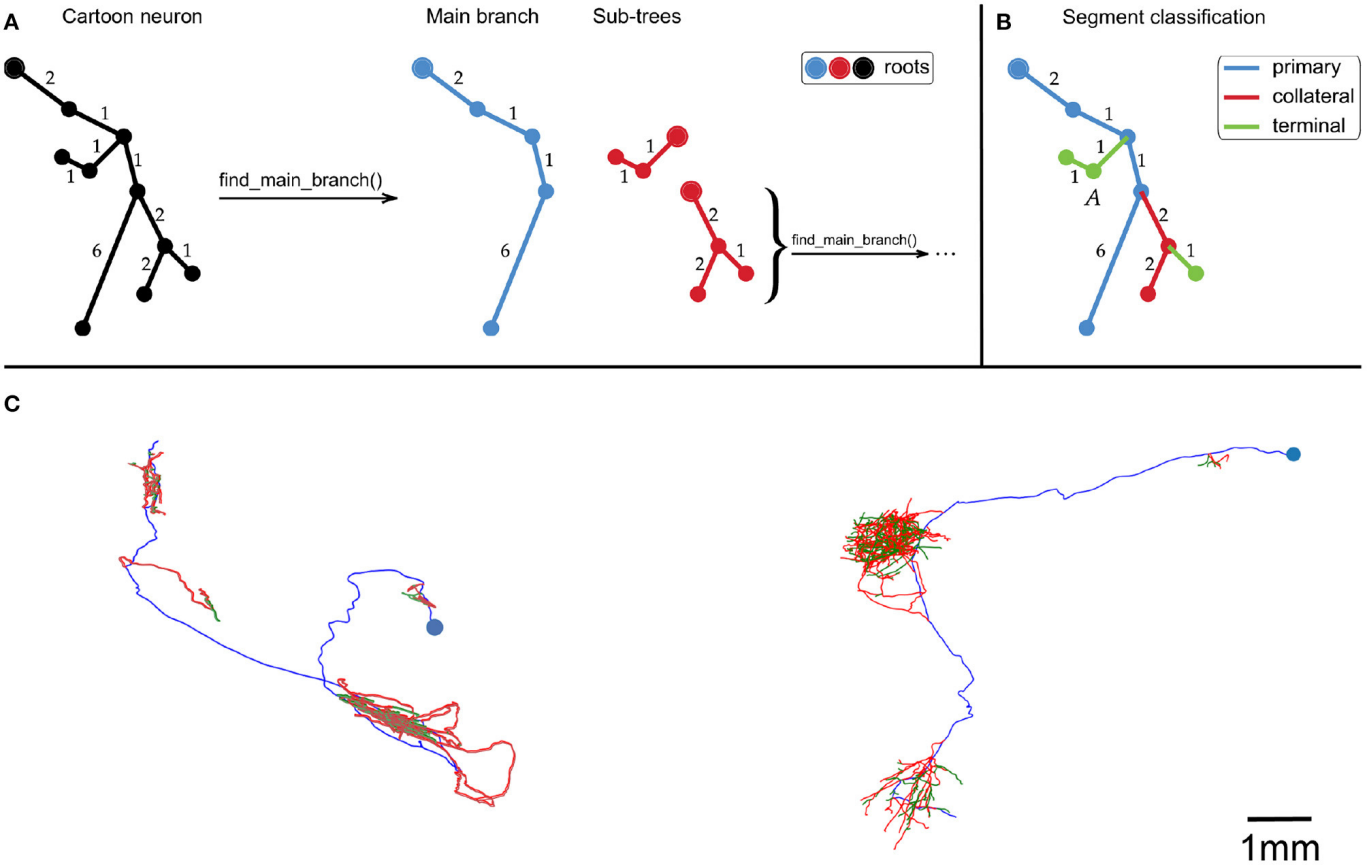
**(A,B)** Cartoon example of how we partition an axonal arbor trace into different segment classes, with numbers indicating distances between points (with arbitrary units). **(A)** A neuron trace is split into different segments by identifying the longest root to leaf path (“Main branch”), and separating sub-trees from it. The sub-trees which still have branch points are processed in the same way until the neuron has been split into segments. By using path length to identify the Main branch, this splitting process is invariant to rigid transformations of the trace. **(B)** Illustration of how axon segments are classified as primary, collateral, or terminal. The first segment is defined as primary, and segments that have no sub-trees are defined as terminal. All other segments are defined as collateral. **(C)** Examples of our spline fitting method applied to neuron traces from the MouseLight project (Winnubst et al., 2019). The splines pass through all trace points, and are thrice continuously differentiable for segments that contain at least five trace points. The blue points indicate the somas, and the spline colors indicate segment class (blue = primary, red = collateral, green = terminal). The neuron on the left is from brain 1, the one on the right is from brain 2. The scale bar only applies to **(C)**.

First, the neuron traces were split into segments by recursively identifying the longest root to leaf path (Figure 1A). The first axon segment to be isolated in this way was defined to be the “primary” segment. Subsequent segments that branched were defined as “collateral” segments, and those that did not branch were defined to be “terminal” segments (Figure 1B). This classification approximates the standard morphological definitions of primary, collateral and terminal axon branches.

Next, a B-spline was fit to each point sequence using scipy's function splprep (Virtanen et al., 2020). Kunoth et al. (2018) provide an in depth description of B-splines and their applications. Briefly, B-splines are linear combinations of piecewise polynomials, sometimes called basis functions. The basis functions are defined by a set of knots, which determine where the polynomial pieces meet, and degree, which determines the degree of the polynomial pieces. The *j*'th basis function for a set of knots ξ and degree *p* is recursively defined by Equation (1.1) in Kunoth et al. (2018):

$$\begin{array}{l} B_{j,p,\xi }:=\frac{x-\xi_{j}}{\xi_{j+p}-\xi_{j}}B_{j,p-1,\xi }(x)+\frac{\xi_{j+p+1}-x}{\xi_{j+p+1}-\xi_{j+1}}B_{j+1,p-1,\xi }(x)\\ \text{ with}\\ B_{i,0,\xi }:=\{\begin{array}{ll} 1, & \text{if }x\in [\xi_{i},\xi_{i+1}),\\ 0, & \text{otherwise}. \end{array} \end{array}$$

Splines are fit to data by solving a constrained optimization problem, where a smoothing term is minimized while keeping the residual error under a specified value (Dierckx, 1982). Here, we constrain the splines to pass exactly through all points in the original trace, which corresponds to a smoothing condition of *s* = 0 in splprep. For a sequence of *n* > 5 points, we fit a spline of degree 5, which is the minimal degree that ensures that the splines are thrice continuously differentiable. Differentiability is important because it allows for estimation of curvature and torsion, explained in the next section.

Sequences of fewer than 5 points, however, required lower degree splines to fully constrain the fitting procedure. For a sequence of 3 < *n* ≤ 5 points we used degree 3, for a sequence of *n* = 3 points we used degree 2, and for a sequence of *n* = 2 points we used degree 1. By selecting the degree in this way, we avoided splines of large even degree, such as fourth order splines, which are not recommended in our interpolation setting (Virtanen et al., 2020). Also, these degree choices are low enough to allow for a fully constrained fitting procedure, but high enough to make curvature/torsion nonvanishing when possible.

We recall that B-splines are not required to be parameterized by the arclength of the curve. Here, we set ξ = {0, …, *L*}, where *L* is the cumulative length of the segments connecting the vertices of the trace, in μ*m*. All other spline fitting options were set to the defaults in splprep. This spline fitting method can be applied to any set of points organized in a tree structure, such as a SWC file. Figure 1C shows examples of splines that were fit to neuron traces.

### 2.2. Frenet-Serret Parameters

An important advantage of B-splines is that their derivatives can be computed in closed form. In fact, their derivatives are defined in terms of B-splines as shown below in Theorem 3 from Kunoth et al. (2018):

**Theorem***For a continuously differentiable b-splineB*_*j,p*,ξ_(·) *defined by indexj, degreep* ≥ 1*, and knot sequence* ξ, *we have:*

$$\frac{d}{ds}B_{j,p,\xi }(s)=p\left(\frac{B_{j,p-1,\xi }(s)}{\xi_{j+p}-\xi_{j}}-\frac{B_{j+1,p-1,\xi }(s)}{\xi_{j+p+1}-\xi_{j+1}}\right)$$

*where we assume by convention that fractions with zero denominator have value zero*.

Curvature and torsion can be easily computed because of this property. For a thrice differentiable curve *x*(*s*) ∈ ℝ^3^ that is parameterized by arclength (i.e., ||ẋ(*s*)|| = 1 ∀*s*), one can compute the curvature (κ) and torsion (τ) with the following formulas:

$$\begin{array}{l} \kappa (s)=||\dot{x}(s)\times \ddot{x}(s)||\\ \tau (s)=\frac{\langle (\dot{x}(s)\times \ddot{x}(s)),\dddot{x}(s)\rangle }{||\dot{x}(s)\times \ddot{x}(s)|{|}^{2}} \end{array}$$

defined with the standard Euclidean norm || · ||, inner product 〈·,·〉, and cross product ×. When curvature vanishes, we define torsion to be zero as well, since the torsion equation becomes undefined. The units of curvature and torsion are both inverse length. In this work, neuron traces have units of microns, so curvature and torsion both have units of (μ*m*)^−1^.

Curvature measures how much a curve deviates from being straight, and torsion measures how much a curve deviates from being planar. Together, these quantities parametrize the Frenet-Serret formulas of differential geometry. These formulas completely characterize continuously differentiable curves in three-dimensional Euclidean space, up to rigid motion (Grenander et al., 2007). Curvature takes non-negative values, but torsion can be positive or negative where the sign denotes the direction of the torsion in the right-handed coordinate system. In this work, we are not interested in the direction of the torsion, so we focused on the torsion magnitude (absolute value).

### 2.3. Data

We applied our methods to a collection of cortical projection neuron axon traces from two mouse brains in the HHMI Janelia MouseLight dataset. The precision of the reconstructions is limited by the resolution of the original two-photon block-face images, which was 0.3μ*m* × 0.3μ*m* × 1μ*m* (Winnubst et al., 2019). Each reconstruction is the consensus of traces by two independent annotators. Winnubst et al. (2019) showed that using two annotators per neuron produced reconstructions that are about 93.7% accurate (in terms of total axonal length). There were 180 traces from brain 1 and 50 traces from brain 2.

After fitting splines to these traces, curvature and torsion magnitude were sampled every 1μ*m* along the axon segments. Sampling every 1μ*m* is the highest sampling frequency that does not exceed the image resolution, so it is an appropriate balance of precision and computational efficiency. We studied curvature and torsion magnitude in two ways, described below in sections 2.4, 2.5.

### 2.4. Computing Autocorrelation of Curvature and Torsion

Our first goal was to identify the length scale at which straight axon segments remain straight and curved axon segments remain curved, so we studied the autocorrelation of curvature and torsion magnitude along the axon segments. For each axon segment, the autocorrelation functions of curvature and torsion were computed along the length of the segment, yielding a collection of autocorrelation functions for each brain. Then, we evaluated whether autocorrelation at a particular lag was significantly higher than 0.3 using a one-sided *t*-test with a significance threshold of α = 0.05. We identified 0.3 as our effect size because correlations higher than 0.3 are generally regarded as “moderate” correlations.

It is worth noting that, by the nature of the spline fitting procedure in Virtanen et al. (2020), “lag” in our autocorrelation functions refers to straight line distances between the trace points, not by the arclength of the resulting curves.

### 2.5. Comparing Axon Segment Classes

Our second goal in the analysis was to compare curvature/torsion between segment classes. First, we estimated each segment's average curvature/torsion magnitude by taking the mean from all points that were sampled on that segment.

In order to compare different segment classes, we developed a paired sample method for testing for differences in average curvature/torsion. Different neurons represented different samples, and the average curvature/torsion of two segment classes (primary vs. collateral, collateral vs. terminal, primary vs. terminal) represented the paired measurements.

Define the random variable *X* as the average curvature/torsion of one segment class and *Y* as the average curvature/torsion of another segment class. Further, say *X* and *Y* are both real valued. Our null and alternative hypotheses are as follows:

$$\begin{array}{l} H_{0}:\mathrm{Pr}[X>Y]=0.5\\ H_{1}:\mathrm{Pr}[X>Y]\neq 0.5 \end{array}$$

We tested these hypotheses using the sign test (Neuhauser, 2011). The test statistic is the number of times that the data point from one sample is greater than its pair from the other sample. A key advantage of the sign test is that it does not require parametric distribution assumptions, such as normality of the data. Also, its null distribution can be computed exactly via the binomial distribution. The two different parameters (curvature and torsion), and the three different segment class pairs constitute six total tests, so we applied the Bonferroni correction to α = 0.05 to obtain the significance threshold 0.0083, which controls the family-wise error rate to 0.05. We conducted one-sided sign tests in all cases.

We also wanted to study whether these results would hold if the traces were perturbed. In particular, since the annotators vary the distance between points in their trace, we decided to randomly remove trace points and repeat the curvature/torsion measurements. Since the traces are tree structures, a trace point can be removed after connecting its child node(s) to its parent node. We produced 20 copies of the original dataset and, in each case, removed every trace point with 10% probability.

## 3. Results

### 3.1. Autocorrelation of Curvature and Torsion

**Figure 2:**
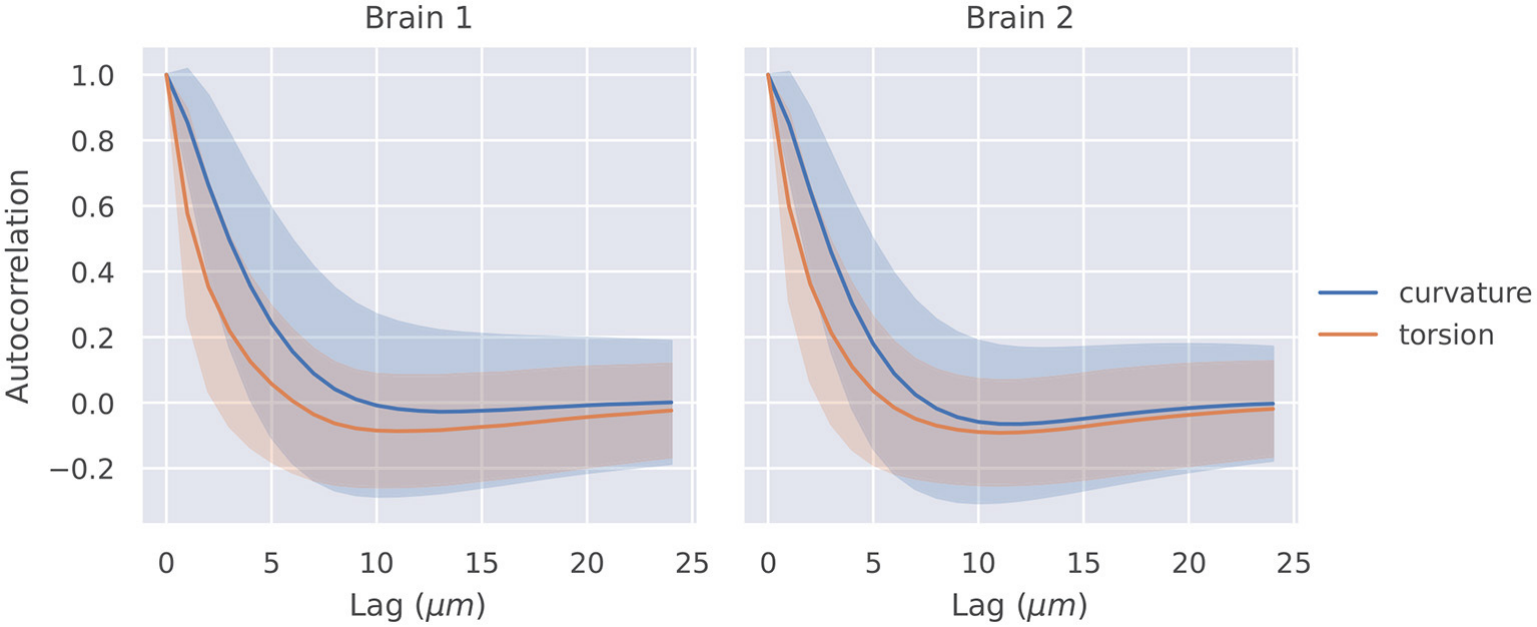
Autocorrelation of curvature and torsion magnitude averaged across all axon segments with ±1σ confidence intervals. Curvature and torsion were sampled at every 1μ*m* along the axon segments. One sided *t*-tests indicated that curvature had statistically significant autocorrelation values above 0.3 at lags of 1, 2, 3, and 4 μ*m* in brain 1 and 1, 2, and 3 μ*m* in brain 2. Torsion had statistically significant autocorrelation values above 0.3 at lags of 1 and 2μ*m* in both brain 1 and 2.

The autocorrelation functions for all segments of a brain were averaged, and they are shown in Figure 2. Also shown is a shaded region that represents one standard deviation of these autocorrelation functions. The *t*-tests described in section 2.4 were significant at lags of 1, 2, 3, 4μ*m* for curvature in brain 1, 1, 2, 3μ*m* for curvature in brain 2, 1, 2μ*m* for torsion in brain 1, and 1, 2μ*m* for torsion in brain 2.

### 3.2. Axon Segment Class Differences

**Figure 3:**
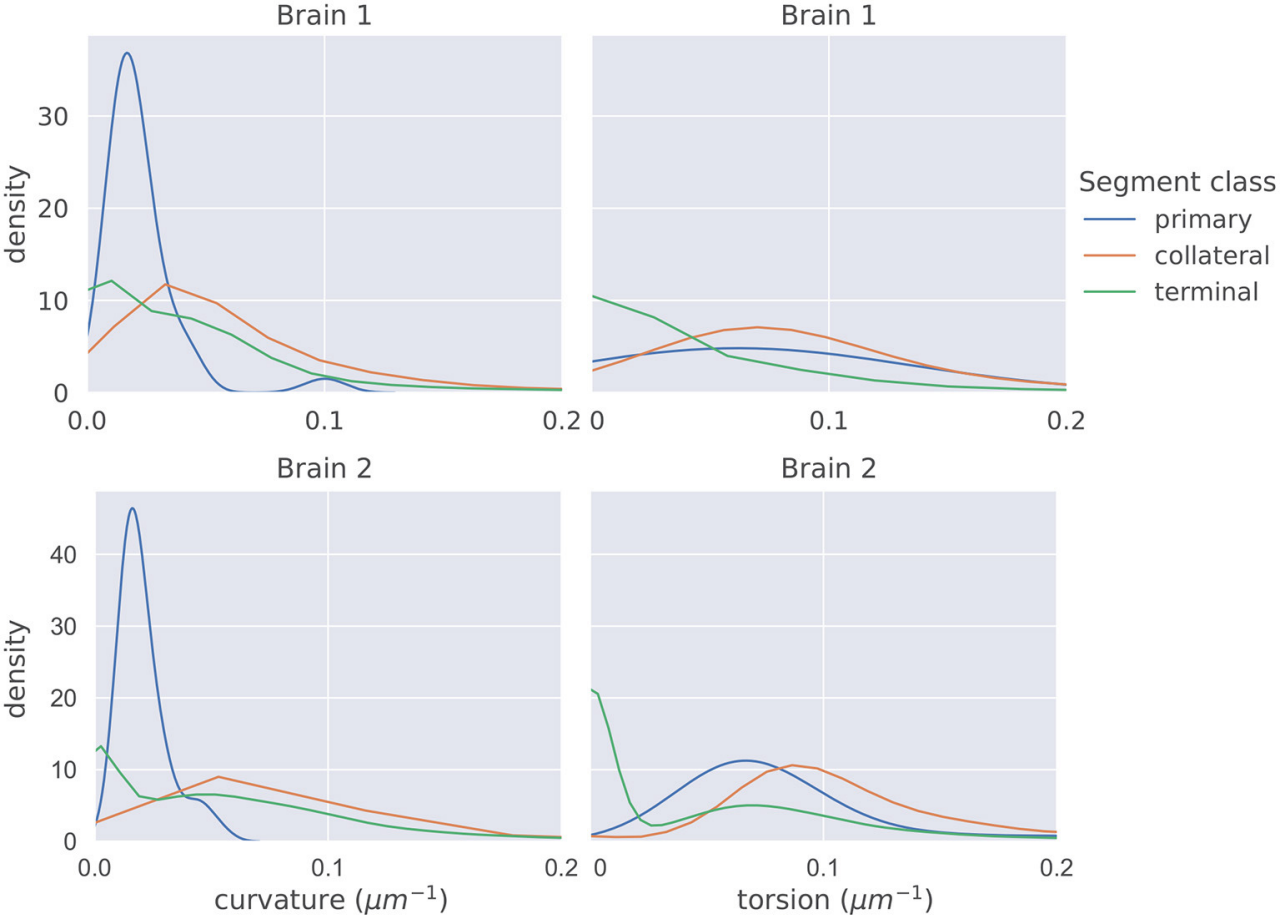
The distributions of average curvature and average torsion differed between the different segment classes as shown in these kernel density estimates (which integrate to one, and therefore density has the units of μ*m*), using a Gaussian kernel. The bandwidth of the kernel was 1.2σ where σ was computed using Scott's method (Scott, 2015). Segment averages were computed by sampling the curves at a uniform spacing of 1μ*m*. One-sided sign tests, testing for differences in average curvature and torsion, were conducted while controlling the family-wise error rate to 0.05. The tests were significant in all cases and the directionality of the tests agreed in both brains.

The distributions of mean curvature and torsion are shown in Figure 3. Our statistical testing procedure, described in section 2.5, rejected the null hypothesis in all cases, with all *p* < 5 × 10^−7^. The directions of the one-sided tests were identical in both brains with:

$$\begin{array}{l} \text{Curvature: Collateral}>\text{Terminal}>\text{Primary}\\ \text{ Torsion: Collateral}>\text{Primary}>\text{Terminal} \end{array}$$

When we applied the same testing procedure to the 20 datasets with trace points randomly removed, the null hypotheses were also all rejected, in the same directions, in all cases.

**Figure 4:**
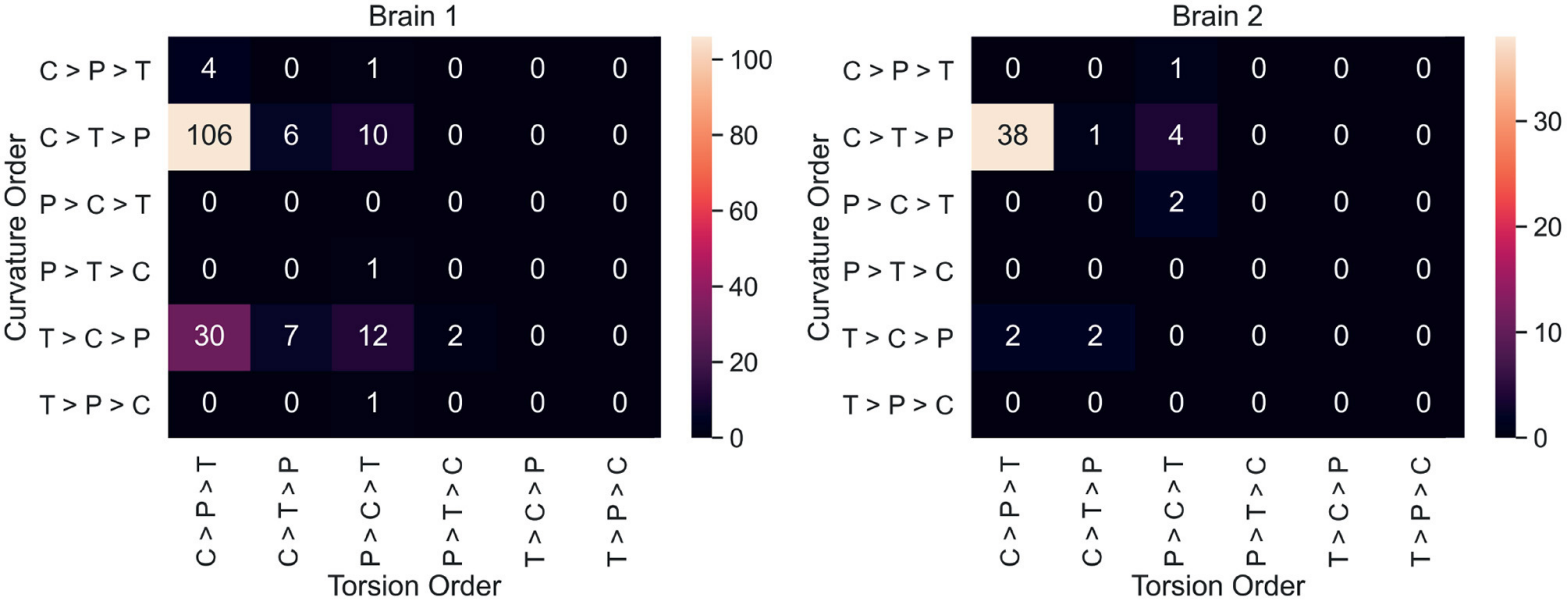
For each neuron, average curvature and torsion was computed for all three segment classes (P, primary; C, collateral; T, terminal) and compared between classes. These heatmaps show the neuron counts for all 36 possible orderings of curvature/torsion. The most common ordering was collateral > terminal > primary for curvature and collateral > primary > terminal for torsion.

Neuron counts for all 36 possible curvature/torsion orderings across classes are shown in Figure 4. The most common ordering of curvature/torsion is exactly the same as the results of the sign test (106/180 neurons followed this ordering in brain 1, 38/50 in brain 2).

In the Supplementary Figure 1, we plot the curvature/torsion vs. segment length. There appear to be modest correlations between segment length and curvature/torsion values in log-log plots.

## 4. Discussion

Our work proposes a model of neuron morphology using continuously differentiable B-splines. From these curves, it is possible to measure kinematic properties of neuronal processes, including curvature and torsion. These techniques are freely available in our open source Python package brainlit: http://brainlit.neurodata.io/, and more information about how to reproduce the specific results here can be found in the data availability statement.

In most contemporary neuromorphological analysis, neuron traces are regarded as piecewise linear structures, which precludes any analysis of higher order derivatives. Our spline representation makes it possible to estimate higher order derivatives and study parameters like curvature and torsion of neuron branches. In the popular piecewise linear representation, curvature and torsion would be zero along the line segments, and undefined where the line segments meet. We simulated a piecewise linear representation by modifying our spline fitting procedure to only produce splines of degree one. Indeed, with this less sophisticated representation, curvature and torsion vanished everywhere, making them not meaningful.

Tortuosity index captures similar information to our curvature/torsion measurements and is popular in neuromorphological analysis (Stepanyants et al., 2004). However, tortuosity requires the user to define start and end points whereas our method does not. Further, the piecewise linear representation of neuron traces limits the sampling frequency of tortuosity. Since tortuosity of a straight line is identically 1, placing the start and endpoints on the same linear segment will always produce a tortuosity value of 1. Our method, on the other hand, can produce more meaningful instantaneous curvature/torsion values.

Our methods for fitting splines and measuring curvature and torsion can be applied in neuromorphological analysis in a variety of ways, but we highlight two applications here, on a dataset of 230 projection neuron traces from two different mouse brains. We found that the autocorrelation functions of both curvature and torsion showed statistically significant correlations above 0.3 within lags of approximately 2 microns (specific lag values given in section 3.1). Next, we defined segments as either “primary,” “collateral,” or “terminal,” and found significant differences in the distributions of curvature and torsion between these classes.

The statistical analysis approach described in section 2.5 satisfies two desirable properties. First, by averaging measurements across segment classes, and pairing the data, we did not have to assume independence between segments of the same neuron. Assuming independence seemed inappropriate because, for example, segments that are connected to each other may have correlated geometry. Second, it avoided any parametric assumptions of the data, such as assuming normality of curvature/torsion measurements. A normality assumption seemed inappropriate for several reasons, including the fact that curvature is nonnegative, and that curvature/torsion was identically 0 for short segments with only 2 trace points.

Figure 4 shows that most individual neurons agree with the overall trend that collateral segments have the highest curvature and torsion. This suggests that the finding here is a consistent phenomenon among projection neurons in mice. In order to explore curvature/torsion distributions one level deeper, we looked into the relationship between curvature/torsion and segment length (see Supplementary Figure 1). In all segment classes, longer segments tend to have less curvature. The relationship between segment length and torsion is weaker, but there does appear to be a positive correlation.

Together, these findings suggest that the geometry of primary axon branches is different than that of higher order branches, such as the segments in terminal arborizations. In particular, higher order branches (collaterals and terminals) had higher curvature than primary branches. Collateral branches also had the highest torsion, but primary branches had higher torsion than terminal segments.

The primary limitation of our work is that our process of splitting a neuron trace into segments may not partition an axonal arbor into the most meaningful segment classes. This is because we needed an unambiguous classification system, while most definitions used in neuroscience literature are subjective and qualitative. For example, collaterals are broadly defined as branches that split off their parent branch at sharp angles, and arborize in a different location from other branches (Rockland, 2013). However, there is no strict cutoff for how far away a branch has to travel for it to be considered a collateral. Further, a branch may be simultaneously considered a collateral and a terminal. We designed a set of segment classes which are mutually exclusive, collectively exhaustive, and agree with common usage of the terms ‘primary,’ ‘collateral,’ and ‘terminal’ by neuroscientists. Future work could include changing our definitions of these classes to incorporate other morphological properties such as branch angle, or axon radius. Also, extending these experiments to neuron trace repositories such as NeuroMorpho.Org would help verify if the results using our classification system generalize.

Previous research has already indicated differences in axon geometry across neuronal cell types. For example, Stepanyants et al. (2004) found higher tortuosity in the axons of GABAergic interneurons vs. those of pyramidal cells. Similarly, Portera-Cailliau et al. (2005) found Cajal-Retzius cells to be significantly more tortuous than Thalamocortical (TC) cells, which is a type of projection neuron. Portera-Cailliau et al. (2005) also offers evidence that, while the primary axon in TC cells travel via a growth cone, most branching occurs via an interstitial, growth cone independent process. Our work elaborates on this distinction, suggesting that higher order axon branches have different geometry as well. While earlier research studied the differences of axonal geometry between neurons, we studied the variation of axonal geometry within neurons.

It is also worth noting that this is not the first work to model neuron traces as continuous curves in ℝ^3^. For example, Duncan et al. (2018) construct a sophisticated and elegant representation of neurons that offers several useful properties. First, their representation is invariant to rigid motion and reparameterization. Second, their representation offers a vector space with a shape metric amenable to clustering and classification. However, their representation is limited to neuron topologies consisting of a main branch and only first order collaterals. Our B-splines approach does not immediately yield vector space properties, but can be applied to neurons with higher order branching, and allows for closed form computation of curvature and torsion. In short, the representation in Duncan et al. (2018) is designed for analysis between neurons, and our representation is designed for analysis within neurons. In the future, we are interested in bringing the advantages of their work to the open source software community, and combining it with the advantages of ours.

This method could also be applied to measure curvature and torsion of dendrites, since dendrites also have a tree structure and are commonly stored in SWC format. However, the segment classes that we define (primary, collateral and terminal) would be inappropriate for dendrites. A segmentation classification system for dendrites would likely depend on the neuron type being studied. For example, a natural classification system of dendrites in pyramidal cells may separate apical dendrites from basal ones while dendrites in Purkinje cells would not have such a division. The researcher would have to define the dendrite segment classes according to the dataset, and the goals of the research.

It is well known that axons are pruned and modified over time (Portera-Cailliau et al., 2005). It is possible that this process contributes to the different geometry of proximal vs. distal axonal segments. Indeed, Portera-Cailliau et al. (2005) mentions the growth of short twisted branches toward the end of axon development. Future animal experiments could follow-up on this idea, and similar experiments to this one could be applied to other neuron types and other species to see if this is a widespread phenomenon in neuron morphology.

## Data Availability Statement

The datasets analyzed for this study can be found in the Open Neurodata AWS account (https://registry.opendata.aws/open-neurodata/). Our package, brainlit provides examples of accessing this data. Specifically, instructions on how to reproduce the figures found here can be found at http://brainlit.neurodata.io/link_stubs/axon_geometry_readme_link.html.

## Author Contributions

MM and DT advised on the theoretical direction of the manuscript. UM advised on the data application experiments. JV advised on the presentation of the results. TA and JT designed the study, implemented the software, and managed the manuscript text/figures. All authors contributed to manuscript revision.

## Conflict of Interest

MM own Anatomy Works with the arrangement being managed by Johns Hopkins University in accordance with its conflict of interest policies. The remaining authors declare that the research was conducted in the absence of any commercial or financial relationships that could be construed as a potential conflict of interest.

## Publisher's Note

All claims expressed in this article are solely those of the authors and do not necessarily represent those of their affiliated organizations, or those of the publisher, the editors and the reviewers. Any product that may be evaluated in this article, or claim that may be made by its manufacturer, is not guaranteed or endorsed by the publisher.

## Supplementary Material

Supplementary Figure 1 The above plots show the relationship between segment length, and mean curvature or torsion in each segment class and brain. Each data point represents a single axon segment, and average curvature and torsion was computed by sampling the segments at a uniform spacing of 1 μ*m*. We removed segments with zero average curvature/torsion in order to plot the data on a log scale. In this data, there appear to be weak negative correlations between segment length and curvature, and a weak positive correlations between segment length torsion.
