# Supplementary figures and images for: Fitting Splines to Axonal Arbors Quantifies Relationship Between Branch Order and Geometry
Source: Front Neuroinform. 2021 Aug 11;15:704627. doi: 10.3389/fninf.2021.704627 (PMC8385655; doi:10.3389/fninf.2021.704627)

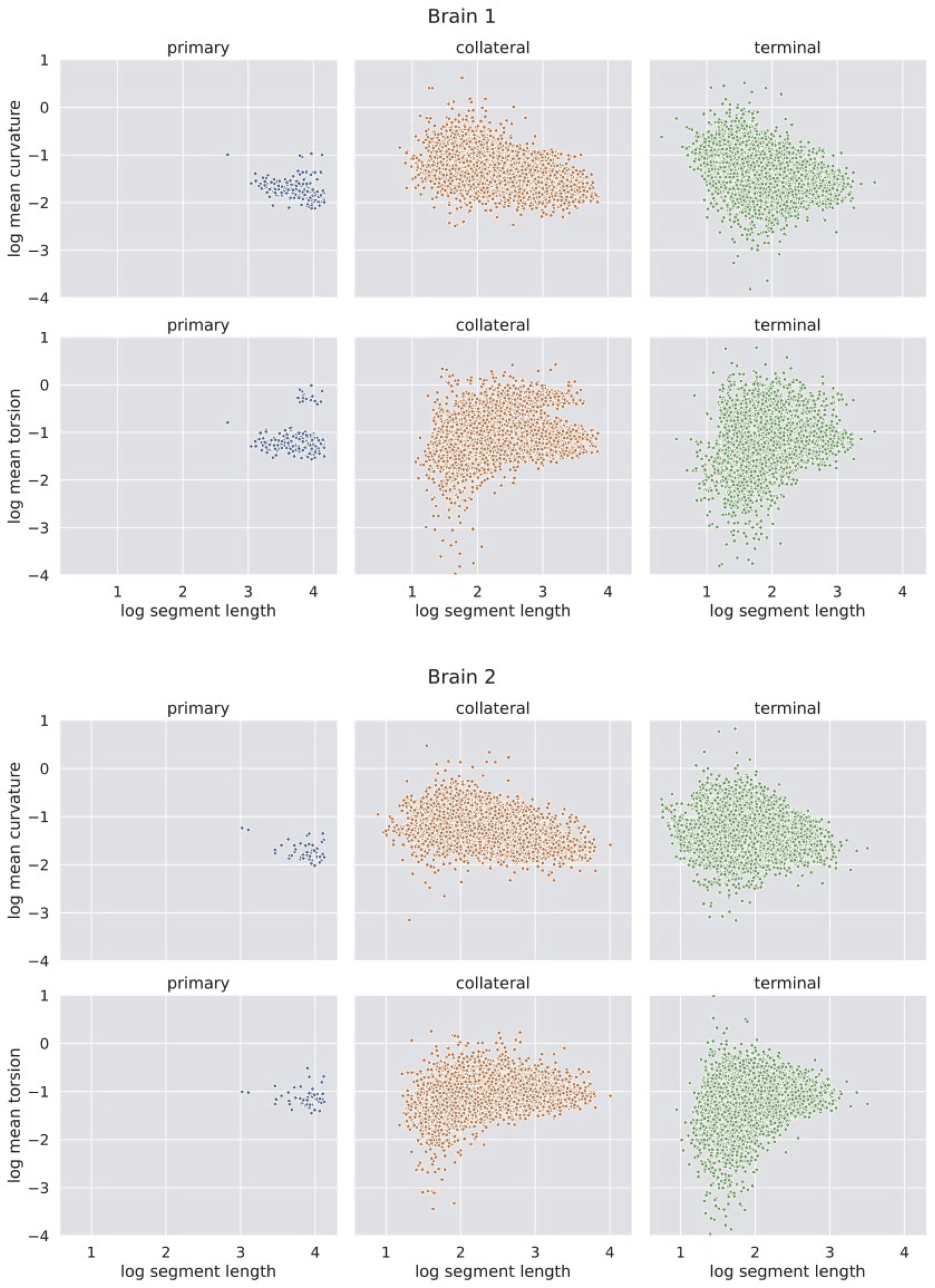

Supplement: Supplementary Figure 1 — The above plots show the relationship between segment length, and mean curvature or torsion in each segment class and brain. Each data point represents a single axon segment, and average curvature and torsion was computed by sampling the segments at a uniform spacing of 1 μm. We removed segments with zero average curvature/torsion in order to plot the data on a log scale. In this data, there appear to be weak negative correlations between segment length and curvature, and a weak positive correlations between segment length torsion. [file Image_1.jpg]
